# Adsorption of Anionic and Cationic Dyes on Activated Carbon Prepared from Oak Cupules: Kinetics and Thermodynamics Studies

**DOI:** 10.3390/ijerph20043280

**Published:** 2023-02-13

**Authors:** Manal Alkhabbas, Alaa M. Al-Ma’abreh, Gada Edris, Tasneem Saleh, Heba Alhmood

**Affiliations:** Department of Chemistry, Faculty of Science, Isra University, Amman 11622, Jordan

**Keywords:** wastewater treatment, adsorption, activated charcoal, oak cupules, naphthol blue black, crystal violet

## Abstract

In this study, activated carbon produced from oak cupules (ACOC) was prepared using chemical activation with H_3_PO_4_. ACOC is subsequently used as an adsorbent to facilitate the removal of an acidic dye, naphthol blue black (NBB), and basic dye crystal violet (CV) from aqueous solutions. The ACOC was characterized by FTIR spectroscopy, XRD, and SEM. The adsorption isotherm data fits well with the Langmuir model for NBB and CV. The kinetic models of adsorption of NBB and CV by ACOC were pseudo-first order and pseudo-second order, respectively. Thermodynamic parameters were evaluated and indicated that the adsorption of both dyes onto ACOC was endothermic and spontaneous. The adsorption capacity of ACOC reached 208 mg g^−1^ for NBB and 658 mg g^−1^ for CV. ACOC was shown to be a promising adsorbent for the removal of NBB and CV from aqueous solutions.

## 1. Introduction

The textile, pulp, and paper industries produce various contaminants, such as dyes and pigments, which are discharged as wastewater. Due to their chemical structure, the dyes are characterized by thermal stability and photostability, and they are also resistant to biodegradation. As a result, they last in the environment for a long time. Dyes absorb and reflect the sunlight that penetrates water, which affects the photosynthesis process of algae and therefore the food chain. Several dyes have been found to be carcinogenic, mutagenic, teratogenic, and toxic to living creatures. Thus, their removal from wastewater is a necessity [1,2].

**Figure 1:**
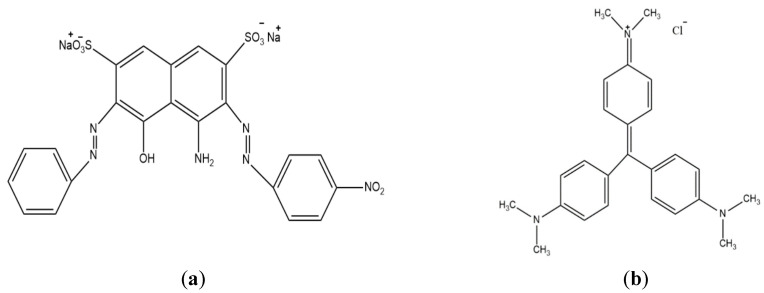
Structure of (**a**) naphthol blue black and (**b**) crystal violet.

Naphthol blue black (NBB) dye (Figure 1a) belongs to the azo group dyes that contain one azo group (-N=N-) at least [3]. NBB dye is classified as an anionic or acidic dye due to the existence of a sulfonate group (-SO_3_^−^). Crystal violet (CV) dye (Figure 1b) is a member of the triphenylmethane dyes, and it is classified as a cationic or basic dye [4].

Several chemical, physical, and biological techniques are suggested for the removal of organic pollutants from wastewater including adsorption, flocculation–coagulation, sedimentation, dialysis, chemical oxidation, ion exchange, membrane separation and biodegradation [5,6]. Adsorption is one of the most effective techniques that is employed for the removal of pollutants from wastewater because it is inexpensive, efficient, simple, and easy to manage [7].

In adsorption, the most widely applied adsorbent is activated charcoal (AC), which is a carbonaceous material produced from wood, coconut shells, soy meal hull, etc. AC is characterized by high porosity and high adsorption capacity. Additionally, it is employed as an adsorbent for the removal of several organic pollutants from air and water. AC was prepared from several types of agricultural waste and was employed for the elimination of different dyes from aqueous solutions [8,9].

The aim of this study is to prepare activated charcoal from oak cupules (cost-free forestry waste) by chemical activation using phosphoric acid. Activated carbon prepared using oak cupules (ACOC) is used to investigate the adsorption capacity for anionic and cationic dyes’ removal, namely NBB and CV, from aqueous solutions. The effects of several factors, such as contact time, adsorbent dose, dye concentration, pH and temperature, are closely examined. The properties of the prepared activated charcoal are analyzed using Fourier transform infrared (FTIR), scanning electron microscopy (SEM), and X-ray diffraction (XRD).

## 2. Experimental

### 2.1. Preparation of the Adsorbate Solutions

The anionic dye (NBB) and cationic dye (CV) were purchased from Sigma-Aldrich. The chemical structures of NBB and CV are shown in Figure 1. The NBB and CV stock solutions of 1.0 g L^−1^ concentration were prepared, and the desired experimental concentrations were made up by diluting the stock solution with distilled water. The pH of solutions was adjusted using 0.1 M HCl and 0.1 M NaOH solutions.

### 2.2. Preparation of the Activated Charcoal

The oak cupules (OC) were gathered from Jerash Province in Jordan, washed with distilled water to get rid of any adhering impurities, and then dried in an air oven at 100 °C for 24 h. This was followed by grinding and sieving to obtain particle sizes less than 0.5 mm. H_3_PO_4_ (85%) was added to oak cupules in a ratio of 3:1 (g H_3_PO_4_/g oak cupules). The resulting slurry was then left overnight, then dried at 120 °C for 4 h. Afterwards, the mixture was heated in a muffle furnace at 450 °C for 1 h. The activated carbon was washed with warm distilled water and a 1.0 M NaOH solution until pH 7, then washed with distilled water. Finally, the prepared ACOC was dried at 110 °C for 6 h.

### 2.3. Characterizations of the Activated Charcoal

The prepared ACOC adsorbent was characterized before and after the adsorption. Scanning electron microscopy (SEM) (FEI Company; Inspect F50 High Vacuum 6 × 10^−4^ Pa, Eindhoven, NB, USA) was used to analyze the surface morphology of the samples. Functional groups of the adsorbent’s surface were determined by FTIR (FTIR-7600 Fourier Transform Infrared Spectrometer, lambda USA) in the wavelength range of 450–4000 cm^−1^. The structural properties of the samples were analyzed using powdered x-ray diffraction (XRD-7000 X-Ray Diffractometer from Shimadzu, Japan) with a nickel-filtered copper radiation (CuKα) and λ = 1.5456 Å; the 2θ range scan was performed from 2° to 60° with a step size of 0.02°.

The pH of the point of zero charge (pH_PZC_) of ACOC was measured by adding 0.05 g of ACOC to a solution of 10^−2^ mol L^−1^ of KNO_3_ with an initial pH of 2–12; the initial pH of the KNO_3_ solution was adjusted using HCl and NaOH solutions. The mixture was stirred for 24 h in a shaker until equilibrium at 25 °C. The final pH values of the solutions were recorded [10,11].

### 2.4. Batch Adsorption Experiments

Adsorption studies of NBB and CV dyes were performed using ACOC as the adsorbent. The batch experiments were conducted using a known quantity of adsorbent and a volume of 100 mL of dye solution at a known initial concentration in an Erlenmeyer flask. The flask was placed in a shaker at 120 rpm and a temperature of 25 °C. The samples were examined at certain time intervals, and the solutions were filtered using a 0.25-μm syringe filter. The concentration of samples was analyzed using UV-visible double-beam spectrophotometer (UV-6100 PC, China) at λ_max_ = 618 and 583 nm for NBB and CV, respectively. The effect of adsorbent dose, reaction time, initial dye concentration, pH and temperature were evaluated. The adsorption capacity, *q_e_* (mg g^−1^), and the adsorption efficiency, *R* (percent of adsorbate adsorbed), were calculated using Equations (1) and (2):

(1) $$q_{e}=\frac{\left(C_{i}-C_{e}\right)}{m_{ACOC}}\times V$$

(2) $$R=\frac{\left(C_{i}-C_{e}\right)}{C_{i}}\times 100\%$$

where *Ci* and *Ce* are the initial concentration and equilibrium concentration of the NBB and *CV* solutions (mg L^−1^), respectively, m_ACOC_ is the mass of adsorbent (g), and *V* (L) is the volume of the solution.

The amount of dye adsorbed at any time, *q_e_* (mg g^−1^), was calculated by Equation (3):

(3) $$q_{t}=\frac{\left(C_{i}-C_{t}\right)}{m_{ACOC}}\times V$$

where *C_t_* (mg L^−1^) is the dye concentration at time *t*.

### 2.5. Isotherm and Kinetic Studies

Analyzing the adsorption isotherm data is important to investigate the interaction between the adsorbent and the adsorbate, and it is necessary to understand the adsorption mechanisms. Three isotherm models (Langmuir, Freundlich, and Temkin) were used to study the behavior of NBB and CV adsorption on the ACOC. The parameters and equations for the selected isotherm models are listed in Table 1. The kinetics of adsorption are employed to investigate the adsorption dynamics. The parameters and equations for the selected kinetic models are shown in Table 1 [12].

### 2.6. Adsorption Thermodynamic

The thermodynamic parameters (Gibbs free energy (*∆G°*), enthalpy (*ΔH°*), and entropy (*ΔS°*) are given by the following equations:

(4) $$\Delta G^{o}=-RTln\left(K_{d}\right)$$

(5) $$\Delta G^{{}^{\circ}}=\Delta H^{{}^{\circ}}-T\Delta S^{{}^{\circ}}$$

where *R* is the universal gas constant (8.314 J·mol^−1^·K^−1^), *T* is absolute temperature in Kelvin (K) and *K_d_* is the apparent adsorption equilibrium constant, and can be determined as follows:

(6) $$K_{d}=\frac{q_{e}}{C_{e}}$$

Δ*H*° and *ΔS°* were computed from adsorption data at different temperatures using the Van’t Hoff Equation:

(7) $$\ln(K_{d)}=\frac{1}{R}\left(\Delta S^{{}^{\circ}}-\frac{\Delta H^{{}^{\circ}}}{T}\right)\;$$

## 3. Results and Discussion

### 3.1. Characterization of Adsorbate

**Figure 2:**
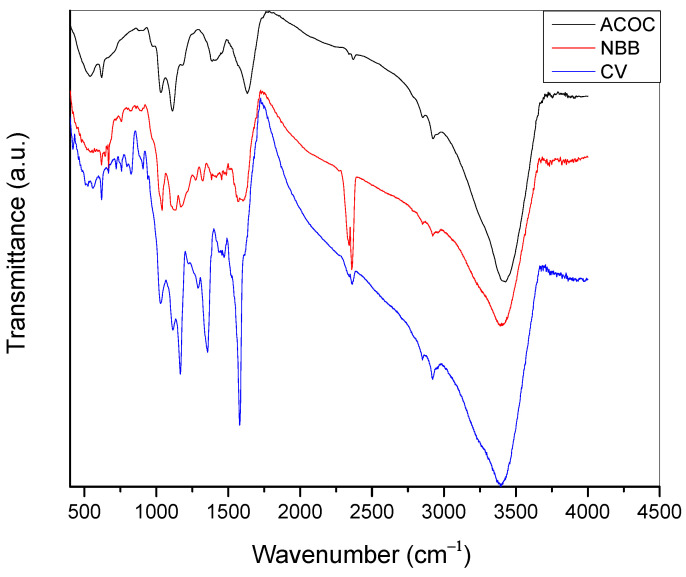
IR spectra of ACOC, ACOC after adsorption of NBB and ACOC after adsorption of CV.

The FTIR spectra of ACOC in the range (4000–500 cm^−1^) before and after the adsorption is provided in Figure 2. The peak at about 3425 cm^−1^ might be attributed to the hydroxyl groups (–OH) of adsorbed water or phenolic group at the ACOC, and this peak is shifted to 3389 cm^−1^ and 3396 cm^−1^ due to the adsorption of NBB and CV, respectively. The shift that occurred might be attributed to the hydrogen bonding that forms between the hydroxyl group on ACOC and the functional groups on the dyes. The peak at 1630 cm^−1^ is attributed to the carbonyl group at ACOC, and it appeared as a broad band and shifted to a lower frequency after adsorption of NBB and CV, respectively. The band at about 1560 cm^−1^ might be attributed to aromatic C═C stretching [13].

**Figure 3:**
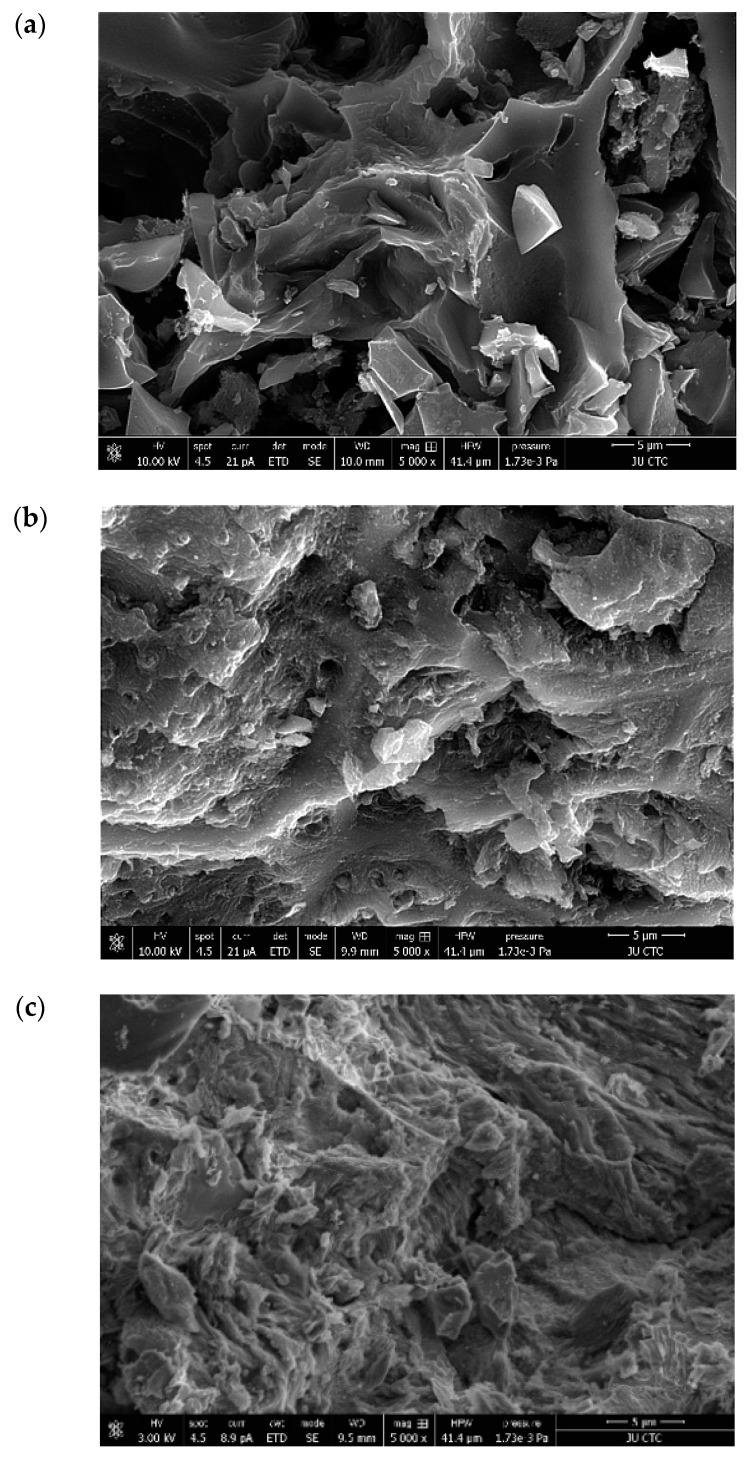
SEM images of ACOC (**a**) before adsorption (**b**) after adsorption of NBB and (**c**) after adsorption of CV.

SEM analysis was employed to study the surface morphology of ACOC before and after the adsorption of NBB and CV. Figure 3a shows the SEM image of ACOC; several pores with varied sizes were observed on the surface of the ACOC. Figure 3b,c display the aggregation of NBB and CV, respectively, on the surface of ACOC.

**Figure 4:**
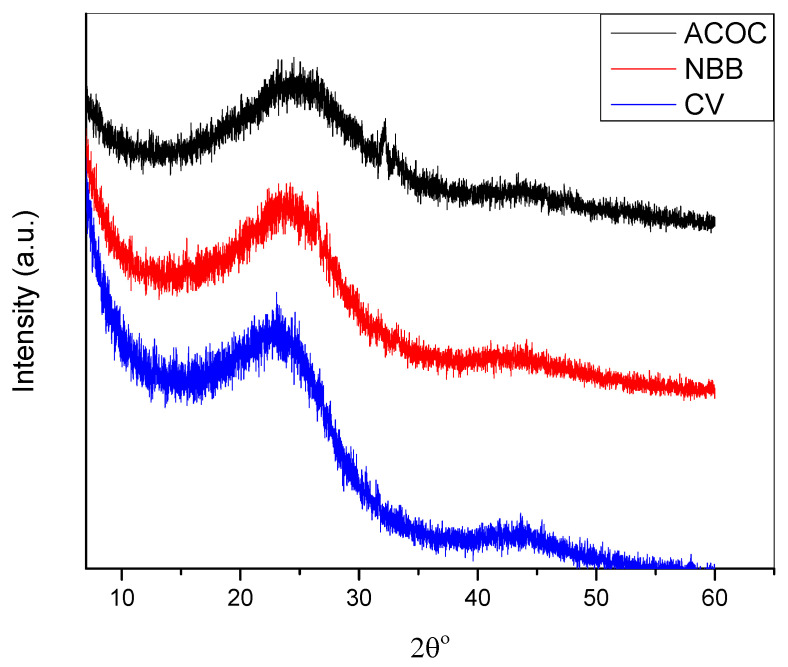
XRD spectra of ACOC before adsorption and after adsorption of NBB and CV.

The XRD patterns of ACOC before and after adsorption of dyes are shown in Figure 4. The broadening in the peaks of the XRD diffractograms is an indication of the amorphous nature of the activated carbon. The position of the peak for ACOC (before and after adsorption) is observed at about 24°, and the result is identical to that obtained in previous studies [14]. An increase in the intensity of XRD peaks is observed after the adsorption of both dyes.

**Figure 5:**
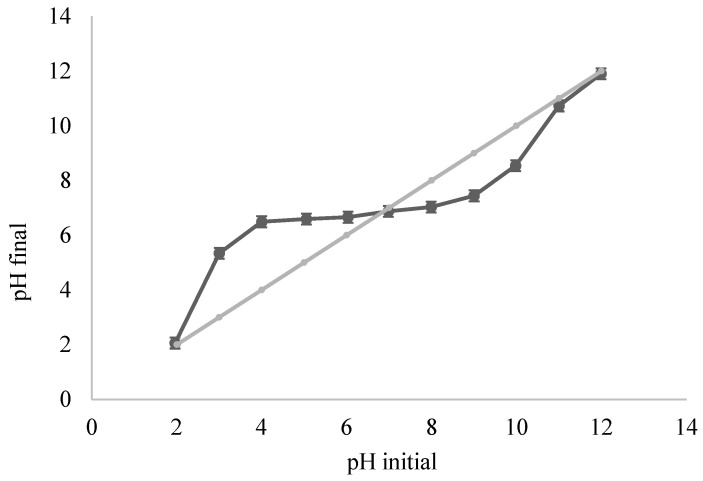
pH_pzc_ determination of ACOC.

The charge and functional groups on the adsorbent surface are affected by the pH of the solution. The pH_pzc_ of ACOC was observed to be 6.96 (Figure 5). At pHpzc, the surface charge of ACOC is zero. As a result, the electrostatic interactions between the positive and negative charges of ACOC are equal [10]. Additionally, it can be observed that the final pH of the solution approaches the initial pH of the solution at pH = 2 and pH = 12; this can be attributed to the high concentration of hydronium ions at pH = 2 and the high concentration of hydroxide ions at pH = 12. So, relatively, a small fraction of hydronium ions and hydroxide ions will be attracted to the negative and positive charges, respectively, on the surface. Thus, a subtle change of pH will occur at high and low values of pH.

### 3.2. Parametric Study of the Adsorption Process

#### 3.2.1. Effect of Contact Time

**Figure 6:**
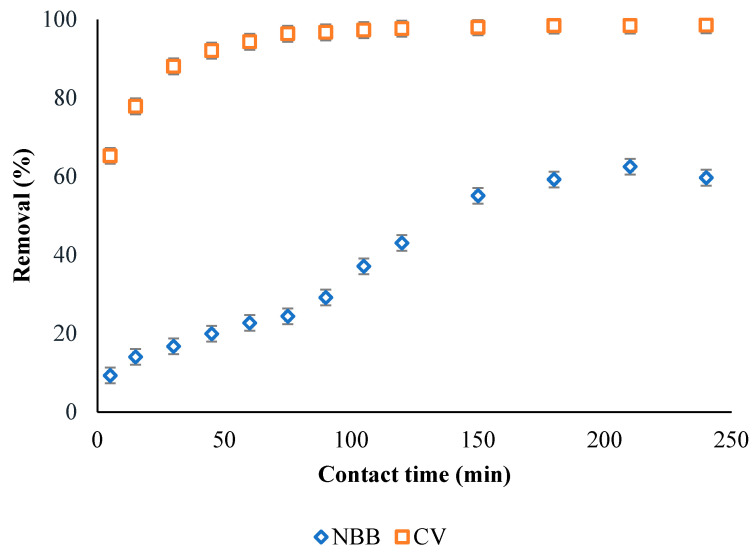
Effect of contact time on the removal of 100 mg L^−1^ initial dye concentration of NBB and CV by 0.3 g L^−1^ of ACOC.

The contact time was investigated up to 240 min, as displayed in Figure 6, with a dye concentration of 100 mg L^−1^, a temperature of 25 °C, and an ACOC dose of 0.3 g L^−1^. The adsorption of both dyes is increased with increasing time until it reaches equilibrium after 180 min for NBB, with 60% removal, and 90 min for CV, with 98% removal.

#### 3.2.2. Effect of ACOC Dose

**Figure 7:**
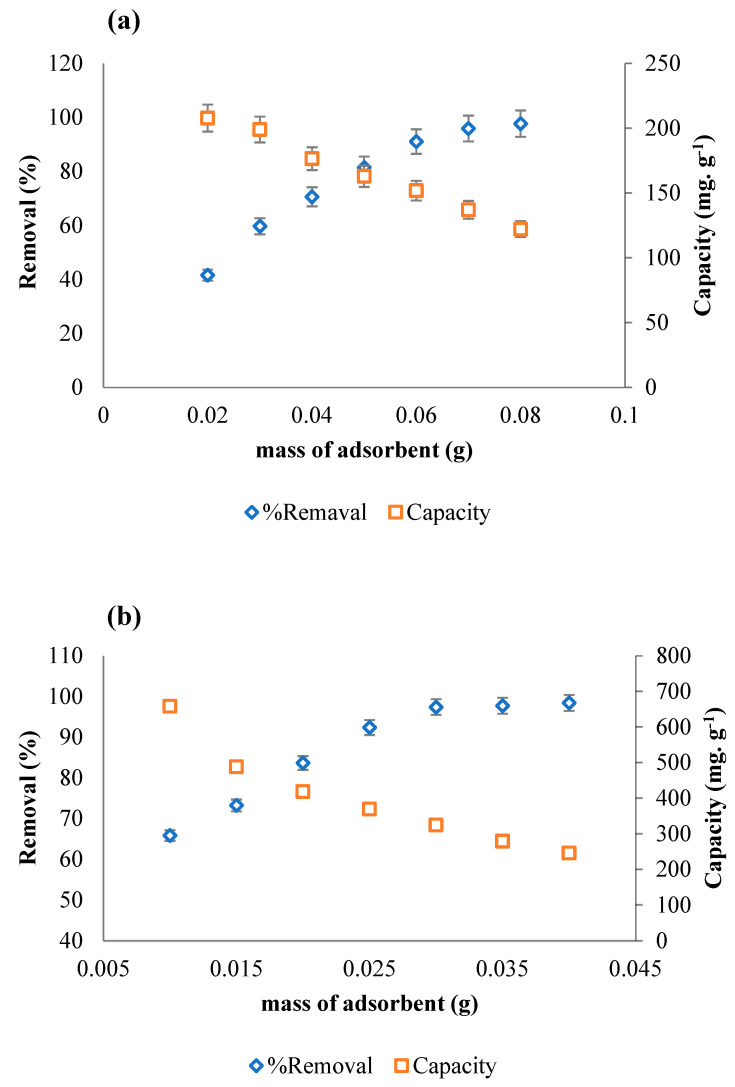
Effect of the ACOC dose on the removal of 100 mg L^−1^ (**a**) NBB and (**b**) CV (T: 25 °C, V = 100 mL).

The ACOC dose was investigated in the range of 0.02–0.08 g for NBB and of 0.01–0.04 g for CV. The dye concentration was 100 mg L^−1^ and the volume of solution was 100 mL. Figure 7 shows the percentage of removal and the adsorption capacity of ACOC (mg dye/g ACOC). The removal percentage was increased from 42% to 98% and from 66% to 98% for NBB and CV, respectively. It is obvious that when the adsorbent dose increases, the removal percentage of the dye increases. This is attributed to the increase in the number of available sites for the adsorption. The adsorption capacity of ACOC reached 208 mg g^−1^ for NBB and 658 mg g^−1^ for CV. The adsorption capacity decreases as the amount of ACOC increases, and this is due to the unsaturation of available sites [15]. The adsorption capacity at 0.02 g of ACOC was 208 mg g^−1^ (0.337 mmol g^−1^) for NBB and 418 mg g^−1^ (1.025 mmol g^−1^) for CV, respectively; thus, the adsorption capacity of particles at the ACOC surface for CV is about three times larger than that for NBB.

#### 3.2.3. Effect of Dye Concentration

**Figure 8:**
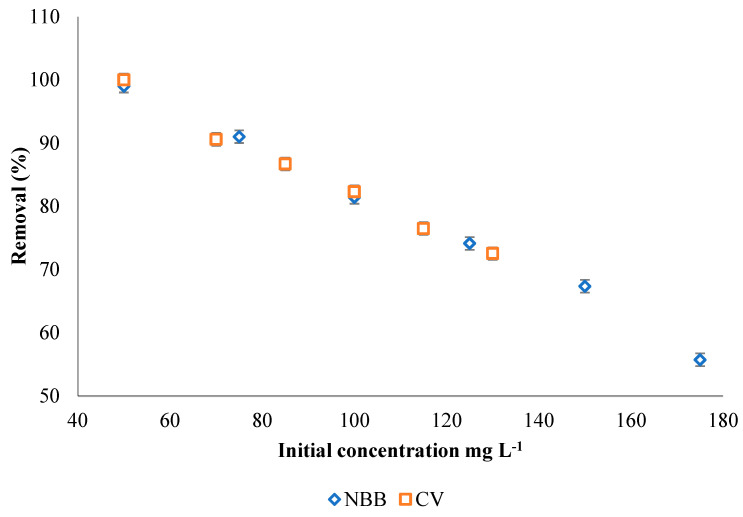
Effect of initial dye concentration of NBB and CV on the adsorption efficiency of ACOC (T = 25 °C, V = 100 mL, m_ACOC_ is 0.050 g for NBB and 0.020 g for CV; contact time is 210 min. for NBB and 120 min. for CV).

The effect of initial dye concentrations was examined in the range of 50–175 mg L^−1^ for NBB (m_ACOC_ = 0.05 g) and 50–130 mg L^−1^ for CV (m_ACOC_ = 0.02 g). As Figure 8 shows, the removal efficiency of both dyes is decreased as the initial concentration of the dyes increases, and this may be attributed to the limited number of adsorption sites that are present on the ACOC surface [7].

#### 3.2.4. Effect of pH

**Figure 9:**
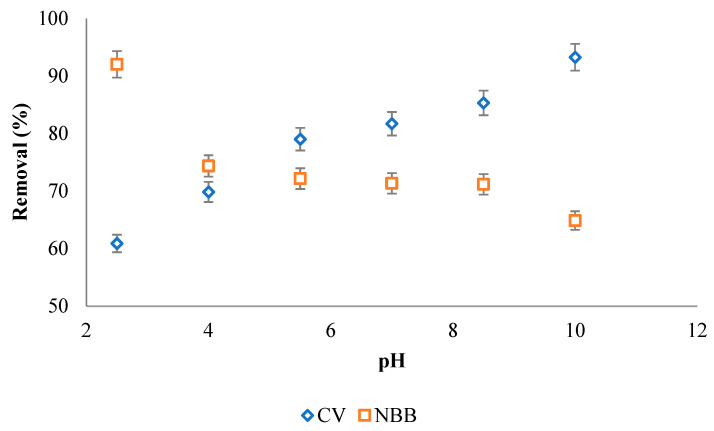
The effect of pH on 100 mg g^−1^ NBB and CV removal efficiency by ACOC (T = 25 °C, V = 100 mL, m_ACOC_ is 0.050 g for NBB and 0.020 g for CV).

The effect of the pH on removal efficiency was investigated at various pH values as shown in Figure 9. For NBB dye, the removal efficiency decreases as pH increases, while for CV dye, the removal efficiency increases as pH increases. At pH < pH_PZC_, the surface of the ACOC has a positive charge and will attract the negative anionic NBB dye species, and at pH > pH_PZC_, the surface of the ACOC has a negative charge and will attract the positive cationic CV dye species. According to previous studies, the removal efficiency for cationic dyes is enhanced when the pH is higher than the pH_PZC_ [12], and the removal efficiency for anionic dyes is enhanced when pH is lower than the pH_PZC_ [16].

#### 3.2.5. Effect of Temperature

**Figure 10:**
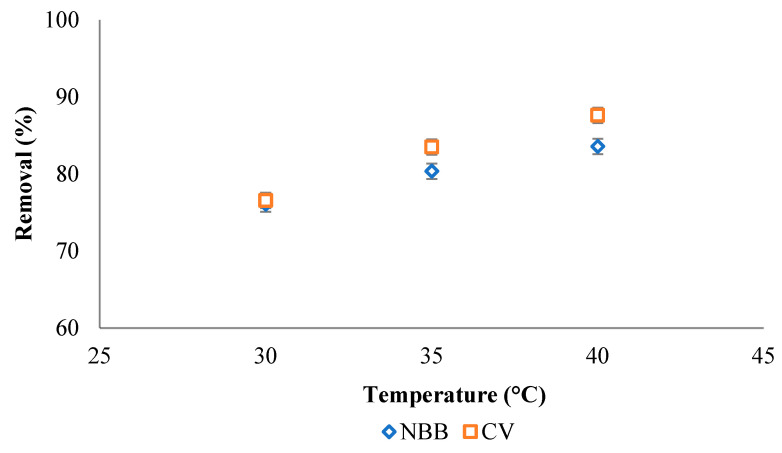
Effect of temperature on the efficiency of removal of 100 mg L^−1^ NBB and CV dyes onto ACOC.

The effect of adsorption temperature is examined at a temperature of 30, 35 and 40 °C for 100 mg L^−1^ of the NBB and CV dyes, as shown in Figure 10. The removal efficiency of both dyes increases as the adsorption temperature increases. The results indicate the endothermic nature of the adsorption reaction for both dyes onto ACOC surface.

### 3.3. Isotherm Studies

**Figure 11:**
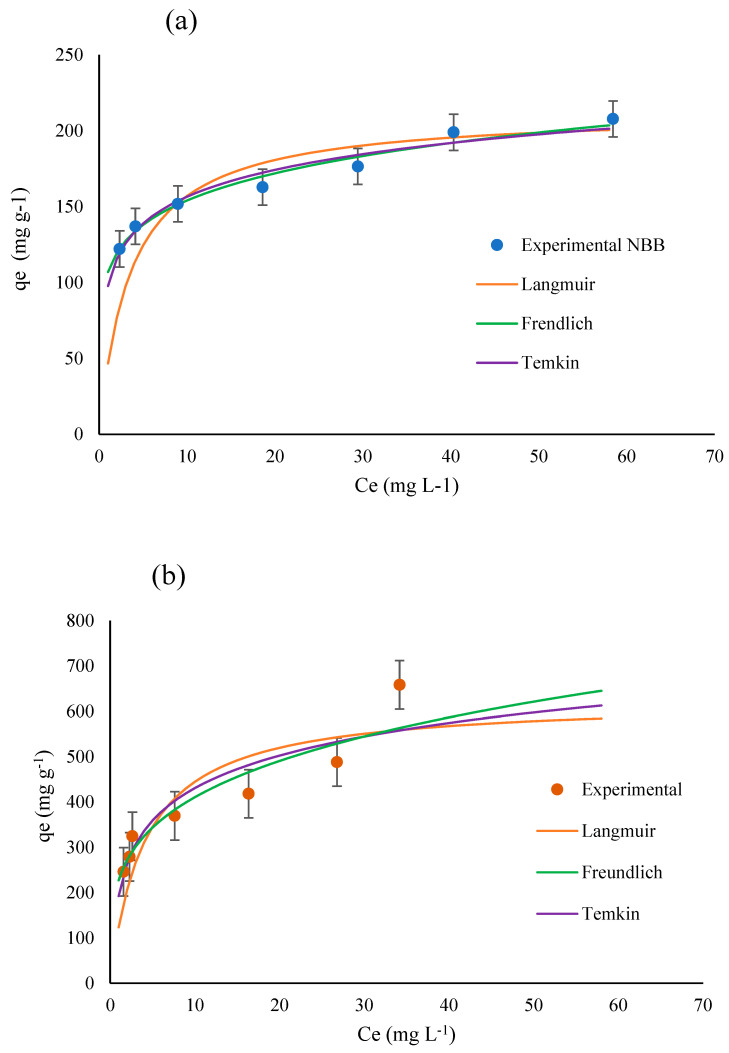
Adsorption isotherm models (Langmuir, Freundlich and Temkin) fitted to experimental adsorption of (**a**) NBB and (**b**) CV.

The mechanism of adsorption for NBB and CV dyes onto ACOC was investigated by Langmuir, Freundlich, and Temkin isotherm models. Table 2 summarizes the achieved results, including correlation coefficients and constants. The Langmuir model introduces the best fit for both dyes, with maximum *R*^2^ for NBB (*R*^2^ = 0.992) and CV (*R*^2^ = 0.935). In the Langmuir model, the *q_m_* value was found to be 213 and 625 mg g^−1^ for NBB and CV, respectively, and these values agree with the experimental ones. Additionally, the *k_L_* value of NBB (0.2814) was larger than that of CV (0.2462), demonstrating that ACOC has greater adsorption energy with NBB than CV [13]. The Langmuir model fit indicates the formation of a monolayer of dye adsorbed at ACOC, with little interaction between the adsorbed species. In the Freundlich model, the values of 1/*n* were 0.1585 for NBB and 0.2572 for CV; this suggested favorable adsorption of both dyes (0.1 < $\frac{1}{n}$ <1) at ACOC [15]. The smallest values for *R*^2^ for both dyes were calculated using the Temkin model. *q_m_* values were 25.5 and 103.5 for NBB and CV, respectively, and these values are far from the experimental values for both dyes. Equilibrium plots of the three isotherm models and the experimental data for the adsorption of NBB and CV onto ACOC are shown in Figure 11.

### 3.4. Kinetics Study

Pseudo-first order, second order, pseudo-second order, and intraparticle diffusion kinetic models were used in this study. The obtained parameters for these models are listed in Table 3. Based on the highest correlation coefficient values, the adsorption of NBB follows the pseudo-first order kinetic model (*R*^2^ = 0.9917), and the adsorption of CV follows the pseudo-second order kinetic model (*R*^2^ = 0.9917). Due to the nonzero intercept, the interparticle diffusion model did not fit with the experimental data for both dyes.

### 3.5. Thermodynamics

**Figure 12:**
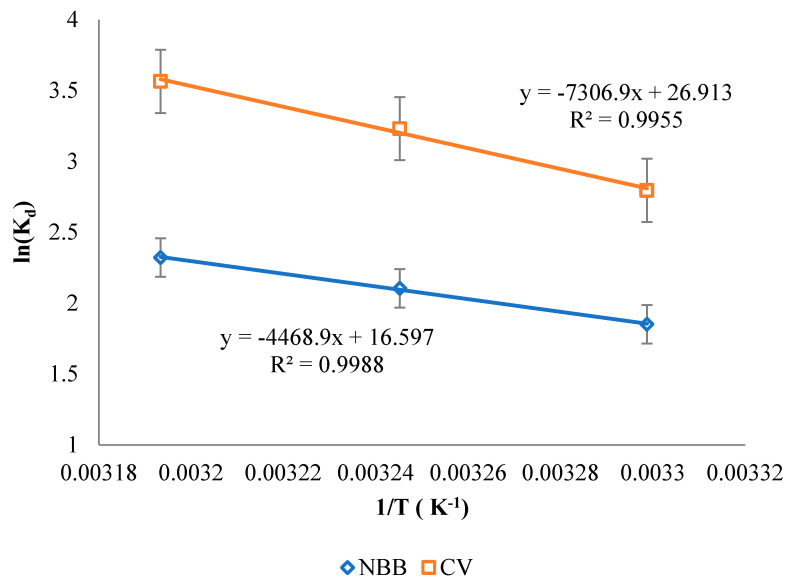
Thermodynamic study for NBB and CV dyes onto ACOC.

The values of *∆H* and *∆S* were determined based on the slope and the intercept from the plot of *ln (Kd)* vs. *1/T* (Figure 12). The values of *∆G* were calculated at different temperatures according to Equation (5). The values of *∆H*, *∆S* and *∆G* are presented in Table 4. Corresponding to the results, all values of ∆G are negative for NBB and CV adsorption, indicating that the adsorption process is spontaneous. Further, the values of *∆G* (kJ/mol) are in the range of 0 > *∆G* > −20, suggesting a physical adsorption of both dyes [4]. The positive values of *∆H* indicate that the sorption process is endothermic. Moreover, the values of *∆S* are positive for both dyes. As a result, *∆G* value decreases as the temperature increases. The positive value of *∆S* can be attributed to the increase in randomness at the solid–solution interface [17], and this is represented by the following reaction:

(Dye).(H_2_O)n_(aq)_ + ACOC_(s)_ ⇌ (Dye).(H_2_O)n-x-ACOC_(s)_ + XH_2_O_(l)_

## 4. Reusability of ACOC

**Figure 13:**
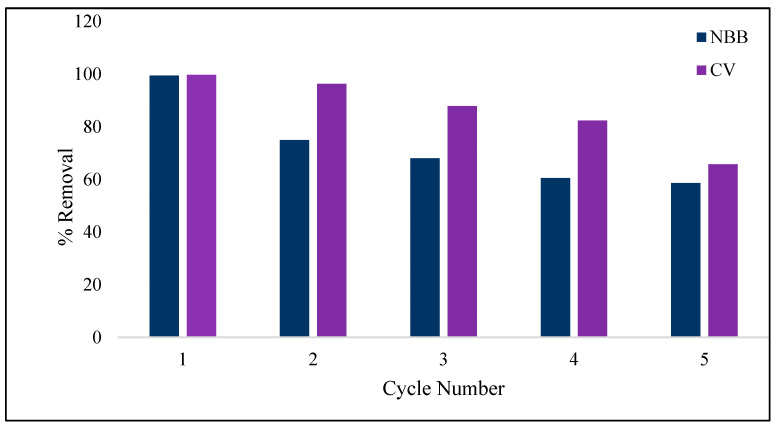
Reusability of ACOC for adsorption of NBB and CV.

The reusability of ACOC was investigated for five cycles of NBB and CV adsorption. Figure 13 illustrates the recyclability of the adsorbent for five cycles. After five cycles, the adsorption efficiency decreases from 99.4% to 58.7% and from 99.7% to 65.7% for NBB and CV, respectively. The decrease in the adsorption efficiency may be attributed to the loss of ACOC during the washing between cycles, as perhaps not all the aggregated dyes’ particles were removed by washing between cycles.

## 5. Conclusions

The activated carbon prepared from oak cupules, a cost-free forestry waste, was effectively used to eliminate anionic naphthol blue black dye and the cationic crystal violet dye from an aqueous solution. The findings obtained for NBB and CV adsorption onto ACOC show that the removal efficiency of cationic dye is better than that of anionic dye. The adsorption data were modelled after a Langmuir isotherm model for both dye; the q_m_ value was found to be 213 and 625 mg g^−1^ for NBB and CV, respectively, and these values indicate high capacity compared to previous studies. The adsorption kinetics of NBB and CV onto ACOC were well fitted using a pseudo-first order model and a pseudo-second order model for NBB and CV, respectively. The adsorption processes of NBB and CV by ACOC were endothermic and spontaneous.

## Figures and Tables

**Table 1:** Equations and parameters of isotherm and kinetics models.

Model	Name	Nonlinear Equation	Linear Equation	Parameters
**Isotherm**	Langmuir	$q_{e}=\frac{q_{m}k_{l}C_{e}}{1+k_{l}C_{e}}$	$\frac{C_{e}}{q_{e}}=\frac{1}{k_{l}q_{m}}+\frac{C_{e}}{q_{m}}$	*C_e_*: Concentration of dye at equilibrium (mg L^−1^) *q_e_*: Amount of adsorbate adsorbed per unit mass of adsorbent at equilibrium (mg g^−1^) *q_m_*: Theoretical maximum adsorption capacity (mg g^−1^) *k_l_*: Langmuir constant related to the rate of adsorption 1/*n* and *K_F_*: Freundlich constants *k_t_*: Temkin constant
	Freundlich	$q_{e}=K_{f}C_{e}^{\frac{1}{n}}$	$lnq_{e}=lnK_{f}+\frac{1}{n}lnC_{e}$	
	Temkin	$q_{e}=q_{m}ln\left(k_{T}C_{e}\right)$	$q_{e}=q_{m}\ln k_{T}+q_{m}lnC_{e}$	
**Kinetics**	Pseudo-first order	$q_{t}=q_{e}\left(1-exp^{-k_{1p}t}\right)$	$\ln\left(q_{e}-q_{t}\right)=\ln q_{e}-k_{1p}t$	*q_t_*: Amount of adsorbate adsorbed per unit mass of adsorbent at time *t* constant (mg g^−1^) *k*_1*p*_: Pseudo-first order rate constant *k*_2_: Second order rate constant *k*_2*p*_: Second order rate constant *k_int_*: Intraparticle diffusion rate constant
	Second order	$q_{t}=\frac{q_{e}}{\left(1+k_{2}q_{e}t\right)}$	$\frac{1}{q_{t}}=\frac{1}{q_{e}}+k_{2}t$	
	Pseudo-second order	$q_{t}=\frac{k_{2p}q_{e}{}^{2}t}{1+k_{2p}q_{e}t}$	$\frac{t}{q_{t}}=\frac{1}{k_{2p}q_{e}^{2}}+\frac{t}{q_{e}}$	
	Intraparticle diffusion	$q_{t}=k_{int}t^{1/2}$		

**Table 2:** Isotherm models parameters for the adsorption of NBB and CV dyes on ACOC.

Model	Parameter	NBB	CV
Langmuir	*q_m_* (mg g^−1^)	213	625
	*k_l_*	0.2814	0.2462
	*R* ^2^	0.992	0.935
Freundlich	*1/n*	0.1585	0.2572
	*K_f_*	106.9	227
	*R* ^2^	0.9768	0.9164
Temkin	*q_m_* (mg g^−1^)	25.5	103.5
	*k_T_*	46.0	6.41
	*R* ^2^	0.9570	0.8475

**Table 3:** Kinetics models parameters for the adsorption of NBB and CV dyes on ACOC.

Model	Parameter	NBB	CV
Pseudo-first order	*K*_1*p*_	0.0173	0.0202
	*q_e_*	125	66.3
	*R* ^2^	0.9917	*R*^2^ = 0.9439
Second order	*k*_2_	−(4 × 10^−5^)	−(2 × 10^−6^)
	*q_e_*	91.7	294
	*R* ^2^	0.571	0.4997
Pseudo-second order	*k*_2*p*_	3.08 × 10^−5^	8.35 × 10^−4^
	*q_e_*	278	333
	*R* ^2^	*R*^2^ = 0.5981	*R*^2^ = 1
Intraparticle diffusion	*k_int_*	0.0861	6.6788
	*R* ^2^	0.9469	0.7128

**Table 4:** Thermodynamic parameters for adsorption of NBB and CV on ACOC.

Dye	ΔH (KJ/mol)	ΔS (J/mol)	ΔG (KJ/mol)		
			303 K	308 K	313 K
NBB	37.15	137.99	−4.68	−5.37	−6.06
CV	60.75	223.75	−11.42	−12.61	−13.80

## Data Availability

The data are available within the body of the article, and any further data can be requested from authors.
